# Refolding and Enzyme Kinetic Studies on the Ferrochelatase of the Cyanobacterium *Synechocystis sp.* PCC 6803

**DOI:** 10.1371/journal.pone.0055569

**Published:** 2013-02-04

**Authors:** Patrik Storm, Tania Tibiletti, Michael Hall, Christiane Funk

**Affiliations:** Deptartment of Chemistry and Umeå Plant Science Centre, Umeå University, Umeå, Sweden; University of Hyderabad, India

## Abstract

Heme is a cofactor for proteins participating in many important cellular processes, including respiration, oxygen metabolism and oxygen binding. The key enzyme in the heme biosynthesis pathway is ferrochelatase (protohaem ferrolyase, EC 4.99.1.1), which catalyzes the insertion of ferrous iron into protoporphyrin IX. In higher plants, the ferrochelatase enzyme is localized not only in mitochondria, but also in chloroplasts. The plastidic type II ferrochelatase contains a C-terminal chlorophyll *a*/*b* (CAB) motif, a conserved hydrophobic stretch homologous to the CAB domain of plant light harvesting proteins and light-harvesting like proteins. This type II ferrochelatase, found in all photosynthetic organisms, is presumed to have evolved from the cyanobacterial ferrochelatase. Here we describe a detailed enzymological study on recombinant, refolded and functionally active type II ferrochelatase (FeCh) from the cyanobacterium *Synechocystis* sp. PCC 6803. A protocol was developed for the functional refolding and purification of the recombinant enzyme from inclusion bodies, without truncation products or soluble aggregates. The refolded FeCh is active in its monomeric form, however, addition of an N-terminal His_6_-tag has significant effects on its enzyme kinetics. Strikingly, removal of the C-terminal CAB-domain led to a greatly increased turnover number, k_cat_, compared to the full length protein. While pigments isolated from photosynthetic membranes decrease the activity of FeCh, direct pigment binding to the CAB domain of FeCh was not evident.

## Introduction

Haemoproteins are an important class of proteins which have diverse biological functions, participating in cellular processes such as respiration, oxygen metabolism and oxygen binding. They are often highly represented and the genome of the annual plant *Arabidopsis thaliana,* for example, encodes as many as ∼400 haemoproteins (Arabidopsis Genome Initiative, 2000). Heme is synthesized in a multistep pathway, 5-aminolevulinic acid (ALA) being the earliest precursor. In plants ALA is used to form tetrapyrroles, which – beside heme production - can also be used in three different pathways, leading to the production of phytochromobilin, the chromophore of the phytochrome family of red/far-red photoreceptors, to sirohaem, the cofactor of nitrite and sulphite reductases and to chlorophyll (Chl), the pigment responsible for harvesting and trapping light during photosynthesis [1], [2]. All tetrapyrroles are synthesized in plastids. The terminal enzyme of the heme biosynthesis pathway is ferrochelatase (protohaem ferrolyase, EC 4.99.1.1), catalyzing the insertion of ferrous iron into protoporphyrin IX.

In mammalian cells ferrochelatase is located in mitochondria, as an integral component of the inner membrane with its active site on the matrix side [3]. Most higher plant genomes, however, contain two ferrochelatase genes, at different locations in the genome [4], [5], [6]. There is no clarity as to whether the different gene products are differentially targeted to chloroplasts and mitochondria [7]. Type I ferrochelatases can be imported into both mitochondria and chloroplasts [6], [8], while type II ferrochelatases specifically have been found to be located in chloroplasts. Reports suggesting their mitochondrial localization have been disputed and the situation still remains unresolved [4], [7], [9], [10]. The unicellular green alga *Chlamydomonas reinhardtii* contains both mitochondria and a chloroplast, but contains only one gene encoding a ferrochelatase, which is homologous to the Type II ferrochelatase found also in photosynthetic cyanobacteria [11].

Type II ferrochelatases of photosynthetic organisms contain a CAB motif, a conserved hydrophobic stretch that corresponds to the chlorophyll-binding domain in the first and third helices of light-harvesting antenna proteins in higher plants [12], [13]. This CAB motif is only present in plant ferrochelatases that are expressed in photosynthetic tissues (Type II), but not in ferrochelatases that are expressed in non-photosynthetic tissues (Type I) [6], [10]. The Type II enzyme is presumed to have evolved from the cyanobacterial ferrochelatase, which also possesses the C-terminal CAB motif [12]. The CAB motif is important for binding of chlorophyll *a* and *b* (CAB) to the higher plant light-harvesting complexes and it is also found in the light-harvesting like proteins (Lil proteins). In the genome of the cyanobacterium *Synechocystis* sp. PCC6803 (hereafter *Synechocystis* 6803), five *lil* genes have been identified, coding for proteins with high similarity to the plant light-harvesting complexes [12]. Four genes encode the small CAB-like proteins (SCPs or high light induced proteins, HLIPs) referred to as ScpB-E, which have a molecular mass of around 6 kDa and have been shown to be involved in chlorophyll biosynthesis and the stabilization of chlorophyll-binding proteins [14], [15], [16], [17]. The fifth gene, also referred to as ScpA, encodes the C-terminal part of the ferrochelatase enzyme. It has been suggested that the ancient ferrochelatase captured a membrane-spanning helix from a SCP/HLIP in order to fulfill functions for membrane anchoring or photoprotection of porphyrins [13]. Changes in the activity of the ferrochelatase have been shown to influence chlorophyll biosynthesis [18], and while inactivation of ScpA only has a subtle effect on enzyme activity [12], truncation of both ScpA and its linker segments impair enzyme activity [19].

Chl is the most abundant tetrapyrrole in plants and cyanobacteria, and the magnesium-chelatase and ferrochelatase enzymes compete for the same substrate, Protoporphyrin IX, for insertion of either magnesium for Chl biosynthesis or ferrous ion for heme biosynthesis, and in cyanobacteria also for phycobilin biosynthesis. However, the control step at the metal insertion branch point is poorly understood. While magnesium-chelatase comprises three subunits, CHLD, CHLI and CHLH [20] and requires ATP for activity, ferrochelatase is composed of a single subunit and requires no cofactors [2]. To guarantee a balanced flow of precursors in the pathway, the distribution of tetrapyrroles to the Fe- or Mg-branch, respectively, has to be tightly regulated. There may be up to 100 times more Chl in a cell than all other tetrapyrroles together [1]. It has therefore been suggested that Chl availability might positively regulate ferrochelatase activity [14], [19]. The expression or activitiy of the chelatases have been studied by various research groups and factors that have been proposed as being important are e.g. ATP-availability, redox state, enzyme localization, gene expression and substrate affinities [6], [21], [22], [23], [24].

In this paper we report a protocol for the functional refolding and purification from inclusion bodies, without truncation products or soluble aggregates, of recombinant *Synechocystis* 6803 ferrochelatase (FeCh). Enzyme kinetics were studied using Zn^2+^ and protoporphyrin IX as substrates for the monomeric form of FeCh that was either refolded from inclusion bodies, co-expressed with chaperones or lacking the CAB domain (FeChΔ347). We elucidated the effect of the C-terminal CAB-domain on the enzymatic activity, and investigated the effect of an N-terminal His_6_-tag.

## Methods

### Production and Refolding of Recombinant Ferrochelatase from Synechocystis 6803 from Inclusion Bodies

**Figure 1:**
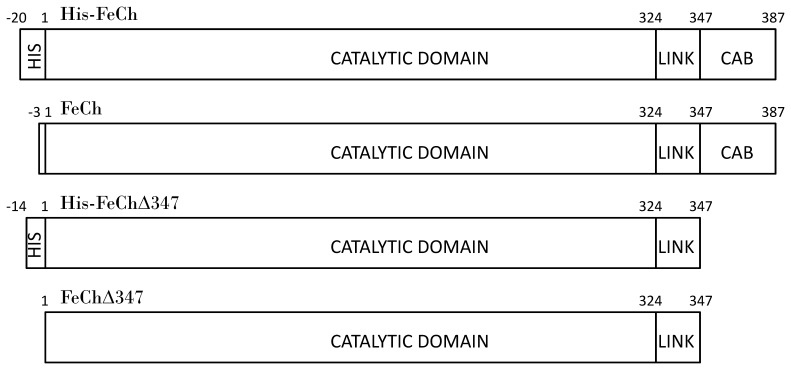
Schematic representation of recombinant His-FeCh, FeCh, His-FeChΔ347 and FeChΔ347 of *Synechocystis* 6803. The C-terminal CAB domain is exclusive to plastidic ferrochelatases of photosynthetic organisms, it is connected via a linker region to the catalytical domain (amino acids 1-324), where chelating of divalent metal ions into protoporphyrin IX takes place. N-terminal His_6_-tags have been added with the amino acid sequence MGSSHHHHHHSSGLVPRGSH (for His-FeCh, cleavable by a thrombin protease) or MAHHHHHHVDDDDK (for His-FeChΔ347, cleavable by an enterokinase), respectively.

The ferrochelatase gene (*hemH*) of *Synechocystis* 6803 (GenBank BAA10523.1) was amplified from genomic DNA using sense primer 5′-GCCGCGCGGCAGCCATATGGGTCGTGTTGGG-3′ and antisense primer 5′-GCTTTGTTAGCAGCCGGACTAAAGCAAGCCGAC-3′, and the PCR product was inserted into the restriction sites Nde I and BamH I in plasmid pET15b (Novagen) using PCR Dry Down Mix (Roche) according to the manufacturers protocol. This resulted in a FeCh construct containing an N-terminal His_6_-tag (His-FeCh, Fig. 1) cleavable by a thrombin protease (amino acid sequence MGSSHHHHHHSSGLVPRGSH). *Escherichia coli* (*E. coli*) strain Rosetta 2 (DE3) was transformed with this plasmid and one litre LB media containing 50 µg/mL arbenicillin and 34 µg/mL chloramphenicol was inoculated with 10 mL over night (o.n.) culture of transformed bacteria and grown at 37°C with shaking at 170 rpm. When the culture reached OD_600_ ∼ 0.5, isopropyl-β-D-1-thiogalactopyranoside (IPTG) was added to a final concentration of 0.5 mM, and growth continued for another 2 hours (or 23°C o.n.). The cells were then harvested by centrifugation. The bacterial pellet was homogenized in 50 mL breakage buffer (0.1 M 2-amino-2-hydroxymethyl-1,3-propanediol (Tris) pH 8, 0.1 M NaCl, 0.2 mM tris (2-carboxyethyl) phosphine (TCEP), 100 µM phenylmethylsulfonyl fluoride (PMSF), 2% (w/v) Triton X-100, 1 mM ethylenediaminetetraacetic acid (EDTA) and 140 000 U lysozyme). After incubation for 30 min at room temperature (r.t., 23°C), 400 U DNAase I were added to the culture together with 1 mM MgCl_2_ and 0.1 mM CaCl_2_. Incubation continued first for 30 min at r.t. and then at 4°C o.n. After centrifugation for 10 min at 12 000×g, the pellet, containing the inclusion bodies, was solubilised in 10 mL solubilisation buffer (50 mM Tris pH 8, 0.5 mM TCEP, 6 M guanidinium hydrochloride (GuA) and 20 mM imidazole) and incubated for 10–15 min at r.t and centrifuged again. The clear supernatant was subjected to Ni-IMAC chromatography using a one mL HisGraviTrap column (GE Healthcare, Uppsala, Sweden) at r.t. in a buffer containing 50 mM Tris pH 8, 6 M GuA and 0.1 mM TCEP. Equilibration of the column had been performed with 10 mL of buffer containing 20 mM imidazole. After loading the supernatant, the column was washed with 5 mL buffer containing 40 mM imidazole. Elution was performed with buffer containing 0.25 M imidazole, collecting one mL fractions. The second IMAC fraction, containing most target protein, was loaded on a Sephacryl S-300-HR size exclusion chromatography column (GE Healthcare) equilibrated with degassed and filtered 50 mM Tris pH 8, 0.1 M NaCl, 6 M urea and 0.1 mM TCEP and run at 0.2 mL/min at r.t. Fractions of one mL were collected from Ve 38 mL to 75 mL.

The fractions containing full length His-FeCh were pooled and refolded on a one mL HisTrap HP column (GE Healthcare) equilibrated with buffer A (50 mM Tris pH 8, 3 M GuA, 0.1 M NaCl and 0.1 mM TCEP). Protein was loaded at 0.3 mL/min flow rate and the column was washed with 5 mL buffer A. Then a 30 mL gradient was applied at 0.3 mL/min towards 100% buffer B (50 mM Tris pH 8, 0.1 M NaCl, 0.5 M KCl, 20% glycerol, 20 mM Na-cholate, 0.1 mM TCEP) and then further washed with 10 mL of buffer B. Refolded and active His-FeCh was eluted by injecting buffer B containing 0.15 M imidazole. To remove imidazole, elution buffer was exchanged to buffer B (without imidazole) over a 5 mL HiTrap desalting column (GE Healthcare) with one mL sample injected; at a flow rate of 2 mL/min protein was collected between 1.5–3 mL. If necessary, the sample was concentrated to 5–10 µM with a VivaSpin500 10 kDa MWCO column (GE Healthcare). Trace metal ions were removed by adding 30 to 50 mg/mL of Chelex-100 (BioRad) to the protein sample and incubating it with intermittent mixing for 1–2 hours at 4°C. Chelex-100 was removed by centrifugation; the supernatant, still at the same protein concentration, was transferred to a new tube. His-FeCh is, based on its activity, stable for at least one week at 4°C or one month at −20°C. Separation of a substantial fraction monomeric protein from higher molecular weight forms was achieved by size exclusion chromatography using a Sephacryl S-100-HR column with buffer B as eluent.

### Chaperone Co-expression of FeCh from Synechocystis 6803

*E. coli* BL21 (DE3) was transformed with plasmid pGro7 encoding GroEL/GroES chaperones (Takara chaperone plasmid set, Takara Bio Inc., Cat.#3340). After subsequent transformation with the pET15b plasmid described above, one litre of LB media supplemented with 50 µg/mL carbenicillin and 20 µg/mL chloramphenicol was inoculated with 10 mL *E. coli* cells from an overnight culture; 0.5 mg/L of L-arabinose was added to induce the chaperone expression. After continued growth at 37°C and 170 rpm shaking, when the culture had reached an OD_600_ ∼ 0.8, transcription of the ferrochelatase gene was induced by adding 0.5 mM IPTG. Cells were harvested by centrifugation after two hours growth and resuspended in buffer B containing 20 mM imidazole. They were broken by sonication, centrifuged and the supernatant was loaded on a HisGraviTrap column equilibrated with buffer B containing 20 mM imidazole. The column was washed with 5 mL buffer B containing 40 mM imidazole and bound proteins were then eluted with buffer B containing 0.15 M imidazole. His-FeCh was treated with Chelex-100 as described above and stored frozen until usage.

### Production of Recombinant His-FeChΔ347 of Synechocystis 6803

The gene for FeChΔ347 (FeCh lacking ScpA and its CAB-domain) was amplified from the FeCh-pET15b plasmid described above using the sense primer 5′–GACGACGACAAGATGGGTCGTGTTGGGGTC–3′ and antisense primer 5′–GAGGAGAAGCCCGGTCTACCATCTTTCCTGGGGATAC–3′. The PCR product was inserted in plasmid pET46 Ek/Lic (Novagen) according to the manufacturers protocol. One litre LB media containing 50 µg/mL carbenicillin was inoculated with an overnight culture of *E. coli* BL21(DE3), which had been transformed with the FeChΔ347-pET46 plasmid and was grown at 37°C with shaking at 170 rpm. At OD_600_ ∼ 0.6 expression of FeChΔ347 was induced by addition of 0.5 mM IPTG to the culture and growth continued over night (∼18 hours) at 23°C with shaking at 170 rpm. Cells were harvested by centrifugation, resuspended in breakage buffer (20 mM Tris pH 8, 30 mM NaCl, 10% glycerol and 20 mM imidazole) and then sonicated on ice. The supernatant after centrifugation contained His-FeChΔ347 with an N-terminal His_6_-tag cleavable by enterokinase (amino acid sequence MAHHHHHHVDDDDK, Fig. 1) and was purified as described for co-expressed His-FeCh.

### Removal of N-terminal His-tag

To remove the N-terminal His_6_-tag, 2.5 mM CaCl_2_ and 40U/mg thrombin (GE Healthcare) (in case of His-FeCh), or 2 mM CaCl_2_ and 20U/mg enterokinase (New England Biolabs) (in case of His-FeChΔ347), were added to the eluted folded protein after IMAC purification and incubated overnight at 23°C. The samples were then diluted with buffer B to an imidazole concentration of 20 mM and purified over a HisGraviTrap column to remove the His_6_-tag as well as uncleaved protein. The unbound material was concentrated by ultra-filtration and purified with a Sephacryl S-100-HR size exclusion chromatography column (GE Healthcare) using buffer B. Fractions containing monomeric FeCh were pooled and concentrated, treated with Chelex-100 and stored at 4°C or used fresh for the enzyme kinetics experiments.

### Preparation of Assay Buffer and Substrates

One hundred mL of assay buffer (0.1 M Tris pH 8.0, 0.1 M NaCl, 0.5 M KCl and 20% glycerol) were degassed, after which 1 mM n-*dodecyl*-β-D-*maltoside* (β-DM) and 0.025% (v/v) Tween 80 were added. Divalent metal ions were removed by filtering the buffer through a column packed with Chelex-100, the first two column volumes were discarded.

Zn^2+^ substrate was prepared from a stock of 0.1 M ZnCl_2_ in MQ-water (a few drops of 37% HCl were added to complete the solubilisation). A dilution series in assay buffer was made to receive the 20 to 200 µM working solutions. Protoporphyrin IX (Proto9) substrate (Frontier Scientific) was prepared in a 1.5 mL tube, 0.5 to 1 mL 0.5% (v/v) Tween 80 (Chelex-100 treated) was added to the powder to receive a 100 µM working solution (ε^408^ = 262 mM^−1^ cm^−1^ in 2.7 N HCl [25]). Zn-protoporphyrin IX (Zn-Proto9, Frontier Scientific) was dissolved similarily to Proto9, its concentration was measured by absorption (ε^400^ = 260 mM^−1^ cm^−1^ in 2% SDS/20 mM NaOH[26]).

### Discontinuous Enzyme Activity Assay

The discontinuous enzyme activity assay [27], [28] was performed by pre-incubating 1.6 µM Zn^2+^ with 57 nM enzyme for 15 min in a test tube at a final sample volume of 125 µL. The incubation temperature was varied as indicated. The assay was started by addition of 0.8 µM Proto9 and stopped after 5 min by adding four volumes of acetone. After centrifugation (13000 rpm for 10 min) the supernatant was transferred to a 2×10 mm cuvette and Zn-Proto9 fluorescence was measured with excitation at 421 nm (slit width 3 nm) and emission spectra collected from 500 to 600 nm (slit width 5 nm).

### Enzyme Kinetic Measurements Using a Continuous Fluorimetric Assay

Zn^2+^ (at a concentration of 0.25 to 10 µM) and freshly prepared monomeric enzyme (30 to 37 nM) were added to assay buffer (1 mL final volume) in a 1×1 cm cuvette and preincubated at 30°C for 5 min while stirring in a Jasco FP-6500 spectrofluorimeter with a Peltier thermostat temperature control. The assay was started by adding Proto9 and a time scan was recorded typically for 150 s with excitation at 421 nm (slit width 1 nm) and emission at 588 nm (slit width 5 nm). The initial rate was calculated from a standard curve with fluorescence at 588 nm as a function of Zn-Proto9 concentration. Solutions depleted of either enzyme, Zn^2+^ or Proto9 were measured as controls to verify that no background signal was present. To avoid deposits of Proto9 on the cuvette walls after each measurement the cuvette was first washed with 0.05% (v/v) Tween 80 and then rinsed with MQ-water.

Of the two substrates (Zn^2+^ and Proto9) one substrate concentration was varied, while the other concentration was kept at an enzyme saturated level. The data were plotted and fitted in Origin 6 (Microcal) to either the Hill equation (when the concentration of Zn^2+^ was varied) or to the Michaelis-Menten equation (when Proto9 concentration was varied). From the fitted data the kinetic parameters K_M_, k_cat_ and V_MAX_ were extracted.

### Pigment Reconstitution

Enzyme (either FeCh, His-FeCh, FeChΔ347 or His-FeChΔ347) was mixed with total pigment extract of *Synechocystis* 6803 as described previously [29] and fluorescence resonance energy transfer (FRET) was measured. Total pigment extract [29] or Chl *a* [29] was also added to assay buffer and incubated for 15–30 min at 30°C or 37°C before the addition of enzyme. After further incubation for 15–30 min FRET and enzyme activity were measured.

## Results

### Expression, Purification and Refolding of His-FeCh from Synechocystis 6803

**Figure 2:**
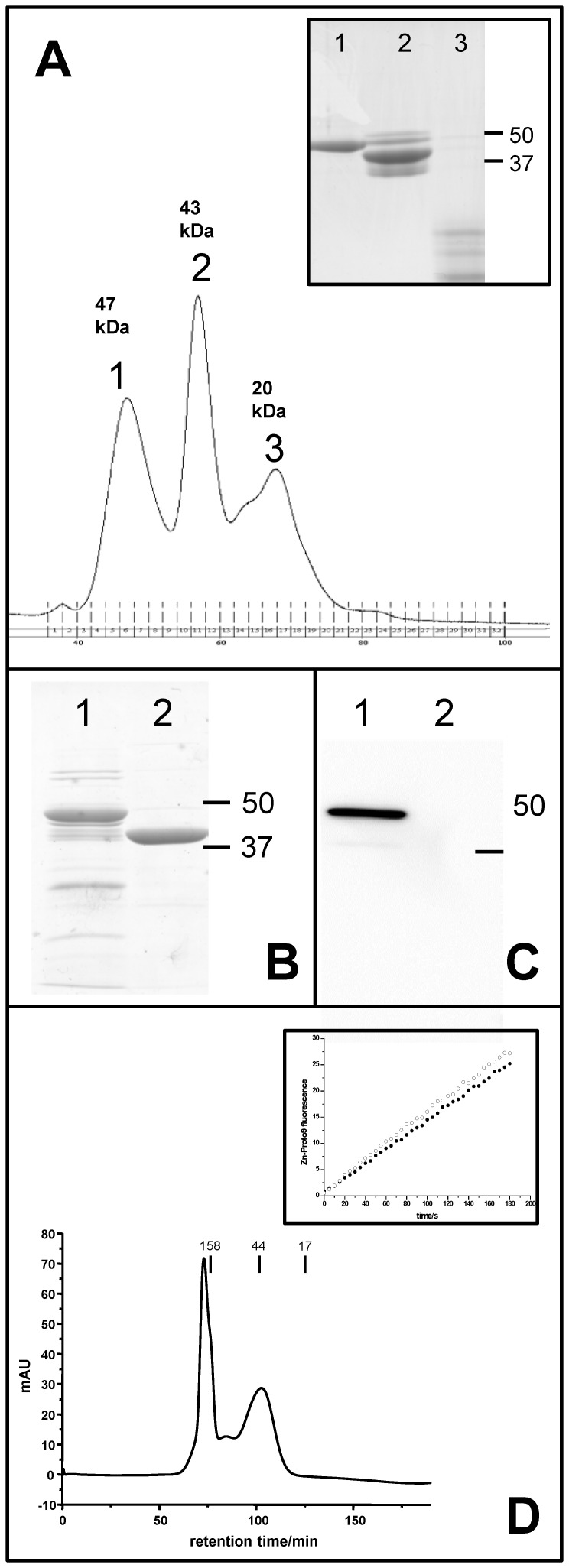
Purification of recombinant His-FeCh or His-FeChΔ347. (A) Chromatogram of size exclusion chromatography at denaturing conditions, separating full length His-FeCh with molecular mass of 47 kDa from truncation products and other contaminants. Inlet: 12% SDS-gel stained by PageBlue (Fermentas) of the elution peaks. Peak numbers in the chromatogram match lane numbers of the inlet. (B) Purification of soluble recombinant His-FeCh co-expressed with GroEL/GroES (lane 1) or His-FeChΔ347 with molecular mass of 41.4 kDa (lane 2). (C) Immunoblot using HRP-conjugated anti-His antibody (Qiagen) detecting the soluble target protein purified from *E. coli*. Lane 1: His-FeCh co-expressed with GroEL/GroES, lane 2: without co-expression with chaperones no recombinant His-FeCh can be immunodetected. (D) Native size exclusion chromatography on full-length FeCh, the monomer has a molecular weight of 44 kDa. Inlet in D: Activity traces of an isolated purely monomeric fraction (filled circles) and a mix of oligomeric states (open circles).

Refolding of proteins *in vitro* is dependent on various factors [30], [31]. Recombinant ferrochelatase of the cyanobacterium *Synechocystis* 6803 was expressed in *E. coli* with an N-terminal His_6_-tag (His-FeCh) in inclusion bodies, and no soluble enzyme could be detected after immunoblotting (Fig. 2C, lane 2). Dialysis at 4°C overnight resulted in active enzyme, however, the sample still contained impurities, i.e. truncation products or soluble aggregates, that were problematic to remove irrespective of the addition of protease inhibitors (Complete from Roche Diagnostics or PMSF). We therefore developed a protocol to separate the His-tagged full length protein with molecular mass of 47 kDa from other proteins by size exclusion chromatography under denaturing conditions (Fig. 2A) before refolding it on a Ni-IMAC column. This approach resulted in a yield of approximately 2 mg folded His-FeCh per liter culture in the absence of truncation products or soluble aggregates. An additive [30] had to be included to receive a substantial fraction of monomeric His-FeCh. In the presence of 0.5 M potassium chloride most protein passed through a 100 MWCO ultrafiltration membrane after refolding [30], and addition of 0.2 M MgCl_2_ or 1% glycine also improved monomerization (data not shown). To improve cryostability and refolding glycerol was added to the solution. Additionally, to solubilize the CAB-domain, which is predicted to form a transmembrane helix, sodium cholate detergent was used, as it has a small micellar size and passes through ultrafiltration membranes. The enzyme in its *in vitro* refolded state, was called “FeCh refolded”. The monomeric form of His-FeCh (and also FeCh) was separated from various oligomeric forms by size exclusion chromatography (Fig. 2D); no difference in activity could be observed between pure monomer and a mixture of monomeric and oligomeric proteins.

In an attempt to purify soluble His-FeCh enzyme, different *E. coli* expression strains (Rosetta2, BL21, C41, Origami), growth temperatures (20°C, 30°C, 37°C) and IPTG concentrations (0.05 mM, 0.1 mM, 0.5 mM, 1 mM) were tested (data not shown). Additionally *E. coli* cells were stressed with 1% EtOH to force chaperone production and also a construct was created, in which maltose binding protein (MBP) was fused to the N-terminus of FeCh. Unfortunately none of these strategies were successful in yielding soluble FeCh protein (data not shown). However, co-expression in *E. coli* with the chaperones GroEL/GroES resulted in a considerable fraction of soluble, mature His-FeCh (1 mg/L culture) (Fig. 2B, C). This folded enzyme was called “FeCh co-expressed”. In order to compare our data with studies performed by others [32] a truncated form of FeCh (His-FeChΔ347), lacking the hydrophobic CAB domain, was also constructed, expressed and purified. Expression of recombinant His-FeChΔ347 did not result inclusion body formation and the soluble enzyme was isolated in its pure monomeric form with a molecular mass of 41.4 kDa (Fig. 2B). To be able to compare the activity of the truncated with the full-length enzyme, assays were performed on the monomeric form of FeCh and/or His-FeCh.

### Influence of Temperature, pH and Buffer Composition on Enzyme Activity

**Figure 3:**
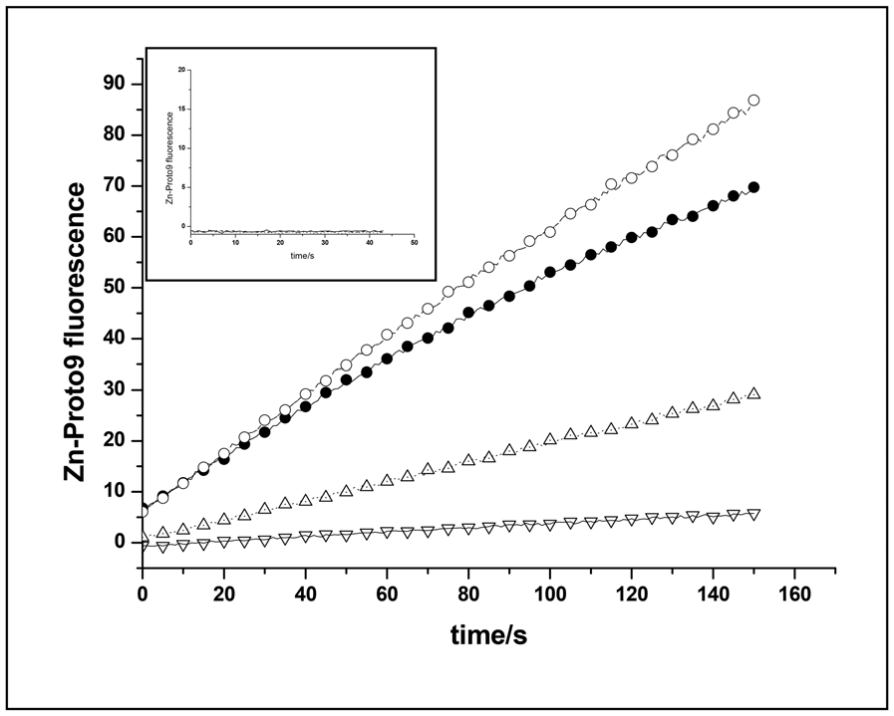
Activity of refolded His-FeCh is dependent on buffer composition. Zn-Proto9 formation was measured at 30°C in assay buffer in the presence of 37 nM His-FeCh, 1 µM Zn^2+^ and 0.5 µM Proto9 (closed circle) using a continuous assay. (Open circle) addition of 0.5 µM Mn^2+^, (open triangle) the detergent β-DM was replaced by 10 mM Chaps, (open inverted triangles) KCl was removed from the buffer. Invoked graph: control activity of standard buffer depleted of His-FeCh, Zn^2+^ or Proto9.

**Figure 4:**
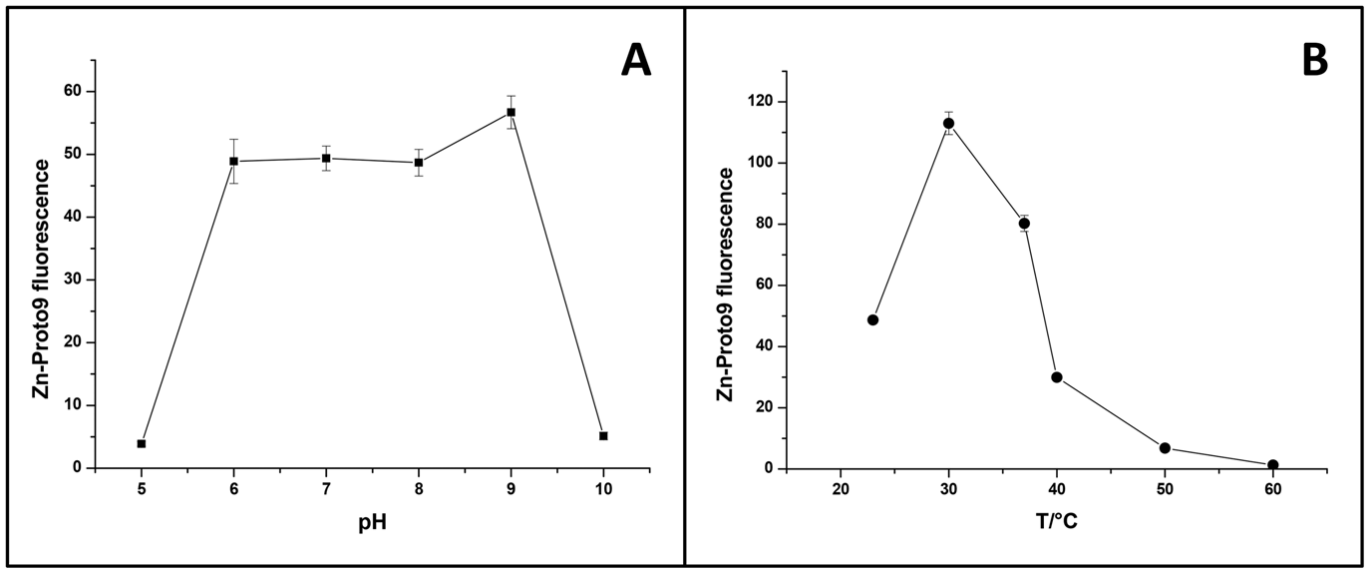
Enzyme characterization using a discontinuous enzyme assay. Effect of pH (A) and temperature (B) were tested, error bars represent standard deviation (n = 3).

Maximal activity was reached when His-FeCh was in its monomeric form, in agreement with ferrochelatase purified from *Synechocystis* 6803 [32], however, a mixure of monomers and oligomers was comparable in activity (Fig. 2D). Buffer composition was chosen for negligible binding of divalent metal ions; Tris proved to be a good choice (Fig. 3) and also is commonly used in similar studies. The buffers 2-(N-morpholino)ethanesulfonic acid (MES) and 4-(2-hydroxyethyl)piperazine-1-ethanesulfonic acid (Hepes) were suitable as well, however, borate as well as Hepes impaired His-FeCh activity at elevated temperatures (37°C) (data not shown). The choice of detergent was important for enzyme activity (Fig. 3), with the usage of CHAPS instead of β-DM leading to a decreased His-FeCh activity. Also the presence of cations was observed as being important for high activity (Fig. 3). Removal of potassium ions reduced the His-FeCh activity considerably while the addition of manganese ions to the standard buffer slightly increased its activity (Fig. 3). Recombinant His-FeCh activity was insensitive to pH variations in the range of pH 6 to 9 (Fig. 4A) and its temperature optimum was at or around 30°C (Fig. 4B), corresponding to the normal growth temperature of *Synechocystis* 6803.

Based on the refolding results and activity data, an assay buffer composed of 50 mM Tris-HCl, pH 8, 0.1 M NaCl, 0.5 M KCl, 20% (v/v) glycerol, 1 mM β-DM and 0.025% (v/v) Tween-80 (maintaining solubility of Proto9) was used in the following measurements. Enzyme activity was typically measured at 30°C.

### Enzyme Kinetics

**Figure 5:**
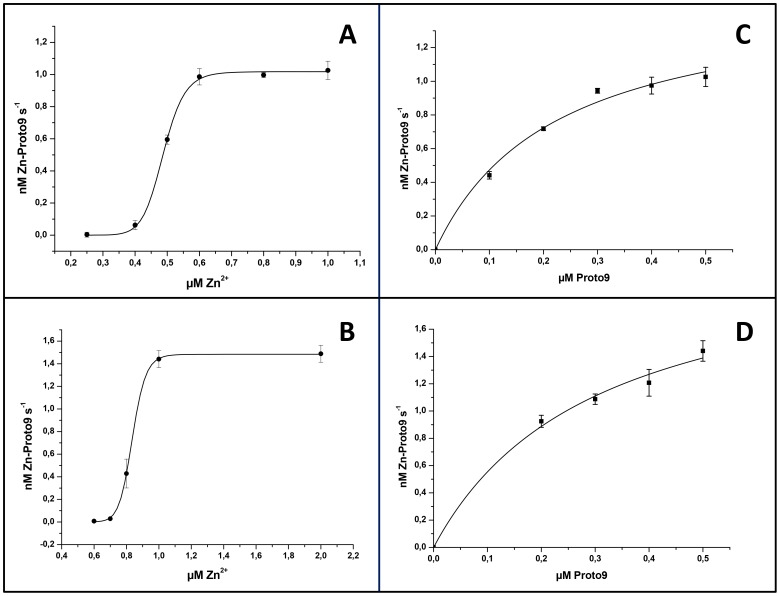
Enzyme kinetic plots for His-FeCh and His-FeChΔ347. 30 nM enzyme was analyzed in a continuous assay at 30°C. Hill equation fit relating initial rate (nM Zn-Proto9 s^−1^) to Zn^2+^ concentration for His-FeCh (A) or His-FeChΔ347 (B). Michaelis-Menten equation fit was used for the dependence of Proto9 concentration on the activity of His-FeCh (C) and His-FeChΔ347 (D). Error bars represent standard deviation (n = 3).

It is well known that ferrochelatases can accept different divalent metal ions and various porphyrin substrates [33]. To be able to compare our data on the recombinant type II FeCh of *Synechocystis* 6803 with activity data observed for orthologues or other ferrochelatases, Zn^2+^ and the natural Proto9 were chosen as substrates. Full-length refolded His-FeCh (Fig. 5 A, C) and truncated His-FeChΔ347 (Fig. 5 B, D) exhibited very similar K_M_ for these substrates. Interestingly both enzymes displayed strong cooperativity regarding the Zn^2+^ substrate (Fig. 5 A-B and Table 1). The midpoint transition varied slightly from batch to batch, but always remained in the sub-µM range (Table 1). The progress curves (measuring product formation over time) of His-FeCh and His-FeChΔ347 had hyperbolic shape when the standard assay was used (as in Fig. 5), but when the detergent β-DM was exchanged against 10 mM Chaps, an initial lag phase of the enzymes became obvious, resulting in a sigmoidal shape. This lag phase could be avoided if the enzymes were pre-incubated with substrate metal for 10 min prior to the assay start (not shown).

**Table 1:** Kinetic parameters K_M_ and k_cat_ and the Hill coefficient *n* of refolded His-FeCh and His-FeChΔ347. Data obtained from Fig. 5.

Enzyme	Zn^2+^			Proto9	
	K_M_/µM	*n*	k_cat_/min^−1^	K_M_/µM	k_cat_/min^−1^
His-FeCh	0.488±0.002	14.7±1.3	2.04±0.02	0.22±0.05	3.1±0.26
His-FeChΔ347	0.836±0.003	20.4±1.2	2.96±0.02	0.30±0.09	4.4±0.60

**Figure 6:**
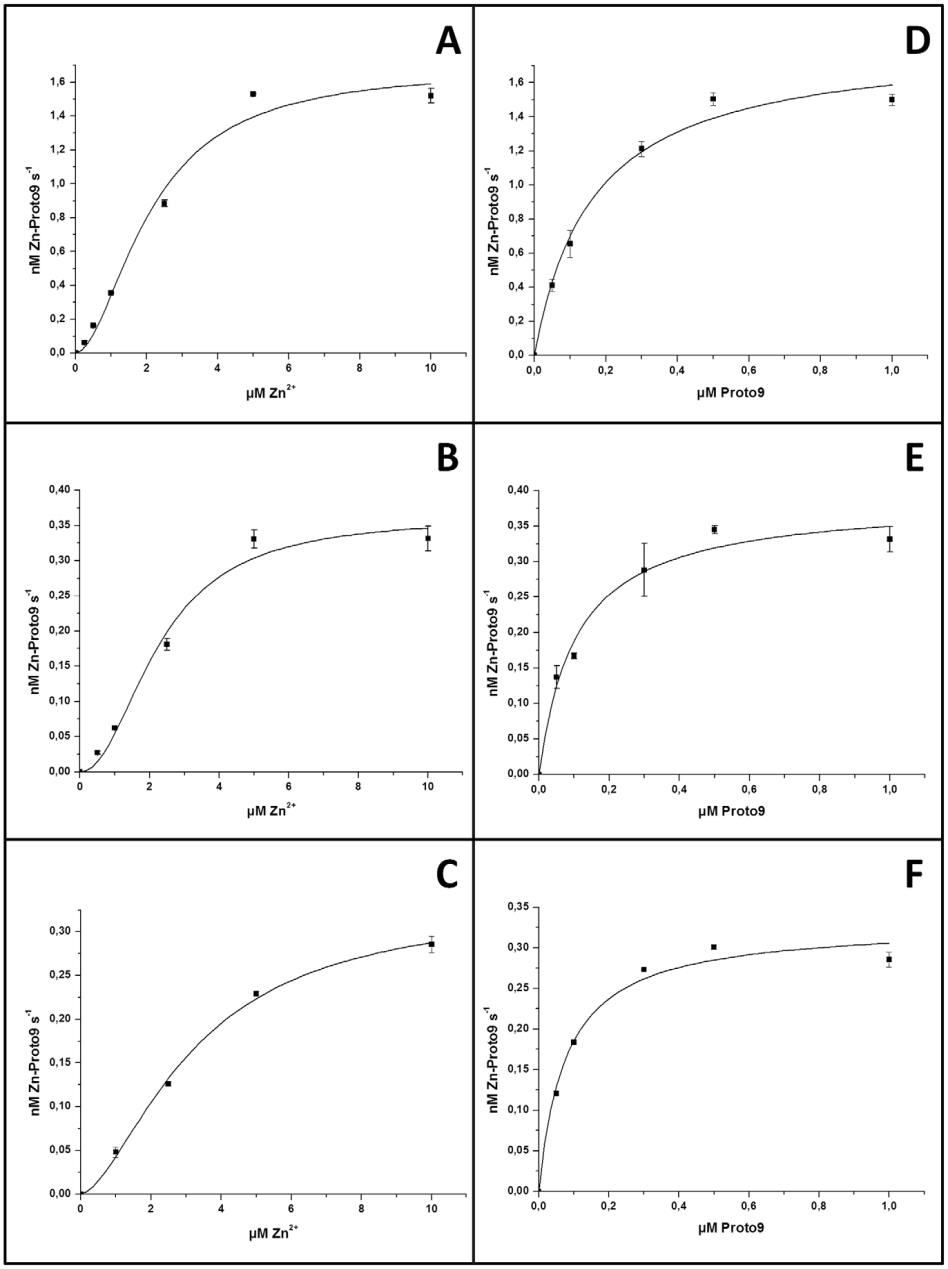
Enzyme kinetic plots for FeCh and FeChΔ347 *lacking* His_6_-tags. 30 nM enzyme was analyzed in a continuous assay at 30°C. Hill equation fit relating initial rate (nM Zn-Proto9 s^−1^) to Zn^2+^ concentration for FeChΔ347 (A), co-expressed FeCh (B) or refolded FeCh (C) to Zn^2+^ concentration. Michaelis-Menten equation fit was used when the variable substrate was Proto9 for FeChΔ347 (D), co-expressed FeCh (E) or refolded FeCh (F). Error bars represent standard deviation (n = 3). “Refolded FeCh” corresponds to *in vitro* folded enzyme, while “co-expressed FeCh” was co-expressed with chaperones assisting folding in *E. coli* cytosol.

Removal of the His_6_-tag lowered the K_M_ of FeCh for Proto9, while the K_M_ for Zn^2+^ was increased and the cooperative effect was less pronounced (Fig. 6 and Table 2). For FeChΔ347 the turnover number k_cat_ was moderately affected by removal of the His_6_-tag, while in contrast removal of the His_6_-tag from the full length FeCh protein resulted in a significant decrease in k_cat_ (Table 2). When FeCh or FeChΔ347, both lacking the His-tag, were pre-incubated in Zn^2+^ containing buffer before the assay was started, the initial activity rate was slightly lower than without pre-incubation. The same effect was observed upon pre-incubation with 0.5 µM Mn^2+^ or 0.5 µM Cu^2+^. Also high Zn^2+^ concentrations (beyond 10 µM) decreased enzymatic activity, indicating substrate inhibition (data not shown).

**Table 2:** Kinetic parameters K_M_ and k_cat_ and the Hill coefficient *n* of *in vitro* refolded FeCh, co-expressed FeCh folded in the presence of chaperones in *E. coli* cytosol, and truncated FeChΔ347, after removal of the N-terminal His_6_-tag. Data were obtained from Fig. 6.

Enzyme	Zn^2+^			Proto9	
	K_M_/µM	*n*	k_cat_/min^−1^	K_M_/µM	k_cat_/min^−1^
FeCh refolded	3.2±0.36	1.6±0.2	0.66±0.04	0.08±0.01	0.66±0.02
FeCh co-expressed	2.2±0.43	2.1±0.6	0.72±0.08	0.11±0.02	0.78±0.04
FeChΔ347	2.1±0.35	1.8±0.4	3.38±0.30	0.16±0.03	3.7±0.22

### Effect of Pigments

Ferrochelatase of photosynthetic organisms is an important enzyme in the regulation of heme-biosynthesis versus Chl-biosynthesis. It has been suggested that the amount of Chl in a cell might regulate ferrochelatase activity, so that common precursors could be used either in one or the other pathway [15], [18]. To test this hypothesis, we therefore performed *in vitro* reconstitution assays on recombinant FeCh or His-FeCh, respectively, with pigments isolated from *Synechocystis* 6803, similar to the experiments performed earlier on the single helix SCPs [29]. Unfortunately all our attempts to reconstitute FeCh or His-FeCh were unsuccessful (data not shown). Pigment-binding should be facilitated in the presence of two transmembrane helices (in analogy to the structure of LHCII, where the first of third helix of LHCII membrane spanning helix are involved in pigment-binding), however, also addition of recombinant ScpD [29] to FeCh did not result in any detectable pigment-binding (data not shown).

**Figure 7:**
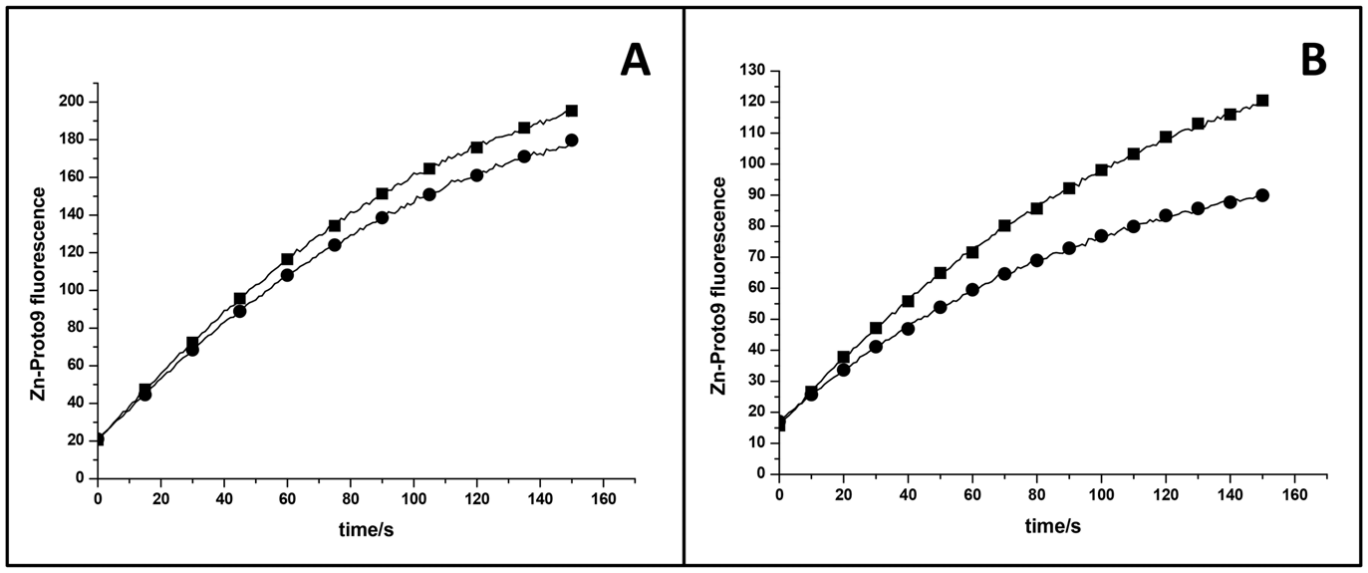
Activity progress curves in dependency of the presence of pigments. Enzyme activity was tested without (closed square) and with (closed circle) pigment mix from *Synechocystis* 6803. Samples in assay buffer at pH 8 were preincubated 30 min at 30°C before the start of the assay. (A) 50 nM co-expressed His-FeCh with 1 µM Zn^2+^, 0.5 µM Proto9 and pigment mix consisting of 0.5 µM Chl and 0.23 µM carotenoids. (B) 30 nM FeChΔ347, 5 µM Zn, 0.5 µM Proto9 and pigment mix consisting of 1.1 µM Chl and 0.5 µM carotenoids.

To test if pigments had any effect on the ferrochelatase activity, the continuous activity assay was performed in the presence of Chl *a* (data not shown) or a pigment mix isolated from *Synechocystis* 6803 (Fig. 7). The presence of pigments resulted in lower enzyme activity of FeCh (Fig. 7A). However, as the activity of FeChΔ347 is similarily affected by the presence of pigments (Fig. 7B), the observed effect seems not to be related to the CAB-domain of the protein.

## Discussion

In this work we present detailed enzymological analyses on a functional refolded monomeric type II ferrochelatase from a photosynthetic organism. We elucidate the effect of the C-terminal CAB-domain on enzyme activity, and investigate the effect of an N-terminal His_6_-tag.

Due to its hydrophobic CAB-domain His-FeCh from *Synechocystis* 6803 was expressed in *E. coli* as inclusion bodies, unless co-expressed with the chaperones GroEL/GroES, while a construct depleted of this CAB motif, His-FeChΔ347, could be expressed fully soluble in *E. coli*. The use of standard purification protocols for purification of His-FeCh resulted either in truncation products or soluble aggregates, despite the usage of specialized *E. coli* strains (e.g. Rosetta2 (DE3)). The site of truncation was located at the C-terminal part of His-FeCh as revealed by immunoblotting and peptide mass fingerprinting (data not shown). We developed a protocol for the purification and refolding of recombinant ferrochelatase in order to circumvent these problems, and here we are able to show that recombinant full-length FeCh of *Synechocystis* 6803 is active as a monomer. In its monomeric state FeCh activity was dependent on the presence of potassium ions to stabilize the native protein structure through an apparent weak kosmotropic effect [30]. Recombinant FeCh activity was found to be insensitive to pH changes in the range of pH 6–9, indicating additional regulation factors *in vivo* for this enzyme in photosynthetic organisms. Type I ferrochelatases are known to be regulated by the redox state of the cell [5], their activity was found to increase in response to environmental stresses, while type II activity is repressed under these conditions [5]. The temperature optimum of FeCh activity was at 30°C, coinciding with the typical growth temperature of *Synechocystis* 6803. At 37°C there was still appreciable activity of the enzyme, which then declined rapidly at higher temperatures (Fig. 4).

The choice of detergent appears to be important for FeCh activity. Attachment to the photosynthetic membranes is required for type II ferrochelatases *in vivo* in order to pursue both uptake of Proto9 and release of heme [27]. β-DM forms oblate micelles mimicking a biological membrane, while CHAPS micelles have a prolate shape [34], [35]. Therefore β-DM seems to be better suited for optimal activity of FeCh compared to CHAPS. The lag phase, resulting in a sigmoidal progress curve that was observed when measuring FeCh activity in the presence of CHAPS, could be abolished by pre-incubating the enzyme with metal ions before the start of the assay. The increased activity after the lag phase therefore was not due to a decreasing zinc pool.

Enzyme kinetic plots revealed cooperativity of FeCh and FeChΔ347 regarding Zn^2+^, the substrate metal therefore might bind to peripheral sites of the enzymes [36], [37]. This cooperativity was even more pronounced studying the His-tagged enzymes (His-FeCh and His-FeChΔ347, respectively). However, the transition in activity was observed at higher substrate concentration than expected by metal binding to the His_6_-tag (1-3 molecules of Zn^2+^ would bind directly to the His_6_-tag). Therefore we assume that the presence of the His-tag affected the entry of substrate into the catalytic cleft [33], [37], [38], as well as the membrane-association properties of the enzyme. The N-terminal domain of the catalytic cleft as well as the CAB-domain have been proposed to be involved in membrane binding of *Synechocystis* 6803 ferrochelatase *in vivo* [32]. Also, Zn^2+^ in solution can cause dimerization of His-tags and therefore influence enzyme activity [39]. Removal of the His_6_-tag from His-FeCh or His-FeChΔ347, respectively, resulted in significant lower affinity for Zn^2+^ as judged by the higher binding constant K_M_. The opposite effect was observed for Proto9.

Studies on the influence of the CAB domain on the activity of the ferrochelatase of *Synechocystis* 6803 have been performed previously [32]. The authors showed that removal of the CAB domain including the linker region inactivates the recombinant protein [19], however, in cyanobacterial crude extracts, removal of the CAB domain only was shown to be dispensable for activity, but important for dimerization [32]. Monomeric and dimeric forms of the enzyme showed similar activities [32]. In our study, the presence of the CAB-domain affected FeCh activity mostly by lowering the K_M_ of Proto9 and the turnover number k_cat_. Strikingly, k_cat_ was much higher for FeChΔ347 than for the full length FeCh. These results are in agreement with data obtained from a study on a *Synechocystis* 6803 FeChΔ347 mutant, which contains more heme, but has a decreased Proto9 pool [32]. Membranes isolated from this mutant have higher ferrochelatase activity than membranes isolated from the wild type [32]. It seems that the CAB-domain of ferrochelatase regulates the Proto9 flux by decreasing its consumption through the heme-branch in favor of the Chl-branch. Indeed, increased amount of chlorophyllide (Chlide) and, less pronounced, of Chl were observed in the FeChΔ347 mutant [32]. However, the exact regulation mechanism is unknown and other molecules interacting with this region might regulate the enzyme activity by changing its structure or orientation. It was speculated that free Chl may bind to the CAB domain and up-regulate its activity [18], [40]. Here, our detailed enzymological studies led to the conclusion that activity is decreased in the presence of Chl *a* or a pigment mix from *Synechocystis* 6803, however this decline of activity seems to be independent of the CAB domain. Binding of Chl to FeCh or FeChΔ347 could not be observed using FRET, as it has been done previously for the other SCPs [29].

Comparing enzyme activity with ferrochelatases from other species, FeChΔ347 is similar to murine ferrochelatase except for higher Proto9 affinity (Table 3). FeCh is similar to yeast ferrochelatase regarding Proto9 affinity, but displays lower metal ion affinity and turnover number. To some extent these observed differences can be explained by variations in assay conditions and other enzyme-specific requirements. Enzyme kinetic parameters of FeCh from *Synechocystis* 6803 have not been estimated before this study. However, FeCh purified from *Synechocystis* 6803 was shown to have a specific activity of 6.5 nmol/min/mg [19], a value comparable to the 17 nmol/min/mg in our studies on refolded or co-expressed recombinant enzymes.

**Table 3:** Enzyme kinetic parameters for ferrochelatases from other species.

Species			k_cat_/min^-1^	Reference
Yeast	0.19–0.25 µM (Zn^2+^)	0.09 µM	23.3	[36], [41]
Murine	1.9±0.3 µM (Fe^2+^)	1.4±0.2 µM	4.1±0.3	[27], [42]
